# Enhancing Children’s Motor Memory Retention Through Acute Intense Exercise: Effects of Different Exercise Durations

**DOI:** 10.3389/fpsyg.2019.02000

**Published:** 2019-08-28

**Authors:** Rosa Angulo-Barroso, Blai Ferrer-Uris, Albert Busquets

**Affiliations:** ^1^Institut Nacional d’Educació Física de Catalunya (INEFC), Universitat de Barcelona (UB), Barcelona, Spain; ^2^Department of Kinesiology, California State University, Northridge, CA, United States

**Keywords:** children, intense exercise, exercise duration, motor adaptation, motor memory consolidation, initial directional error

## Abstract

Physical exercise has been proposed as a viable means to stimulate motor learning. Exercise characteristics, including intensity and duration, may play a role in modulating the exercise effect on motor learning. While some evidence exists regarding the benefits of intense and relatively long exercise, little is known about the effect of short exercise bouts on motor learning, especially in children. This study aimed to assess the effect of long versus short intense exercise bouts on the adaptation and consolidation of a rotational visuomotor adaptation task. The participants were 71 healthy children from two sites divided into three groups: long exercise bout (LONG), short exercise bout (SHORT), and no exercise (CON). Children performed a rotated (clockwise 60° rotation) motor task on four different occasions: an adaptation set and 1 h, 24 h, and 7 days delayed retention sets. Exercise bouts were performed prior to the adaptation set. Results showed a group effect during motor adaptation [*F*(2,68) = 3.160; *p* = 0.049; $\eta_{\text{p}}^{2}$ = 0.087], but no statistical differences were found between groups. Regarding retention tests, both exercise groups (LONG and SHORT) showed superior retention compared to CON group [*F*(2,68) = 7.102; *p* = 0.002; $\eta_{\text{p}}^{2}$ = 0.175]. No differences were found between exercise groups, indicating similar benefits for the two exercise interventions. Overall, whether the exercise duration was long or short, exercise improved motor memory retention as an estimate of memory consolidation process. The use of short exercise bouts may be suitable to improve children’s motor memory consolidation in environments where time constraints exist.

## Introduction

Execution of motor skills involves the integration of perceptual information and motor responses. Learning to adjust a movement to a new mapping between the perceptual and motor components requires a two-phase process, involving adaptation and consolidation (memory formation) of the learned task. During these processes, an initial internal model of the task execution is formed and refined (adaptation) and becomes more stable with the passage of time (consolidation) (Krakauer et al., 2005). Formation of the task’s internal model allows for effective use of feedforward mechanisms, which in turn facilitate the prediction of the sensory consequences of the movement execution (Krakauer and Shadmehr, 2006). Therefore, internal model formation and feedforward mechanisms are key factors in the effective planning of the required movements to fulfill the task objectives (Contreras-Vidal, 2006).

Recent research suggests that exercise potentially protects motor memory formation from external interferences during consolidation (Rhee et al., 2015) and that adaptation and retention can be enhanced by a single bout of exercise in adults (Taubert et al., 2015) and in children (Lundbye-Jensen et al., 2017; Ferrer-Uris et al., 2018). Previous adult research (Roig et al., 2012; Thomas et al., 2016a, b) proposed that exercise characteristics (e.g., exercise timing, intensity, and duration) play an important role in the modulation of the exercise-induced benefits on motor adaptation and memory consolidation.

Exercise intensity and its timing seem to be important modulators in the exercise-motor learning relation for adults. It has been suggested that moderate intensity exercise presented before the practice of the task facilitates motor adaptation (Statton et al., 2015; Snow et al., 2016). On the other hand, many studies have found improvements in motor memory consolidation when an intense exercise (IE) bout has been presented either before or after the adaptation of the motor task (Roig et al., 2012; Mang et al., 2014, 2016; Ferrer-Uris et al., 2017).

Despite the increasing evidence in adults, little is known about the exercise-motor learning relation in children. To our knowledge, only two studies have addressed exercise effects on children’s motor learning. Lundbye-Jensen et al. (2017) found that IE enhanced motor memory consolidation when it was presented after the acquisition of a motor skill, while they did not test the effects of exercise on motor acquisition. Ferrer-Uris et al. (2018) also observed that IE enhanced motor memory consolidation. In addition, they found a stronger effect when exercise was performed immediately before compared to after the motor adaptation presentation. However, Ferrer-Uris et al. (2018) observed that IE had no effect on motor adaptation.

Previous studies have examined how changes in exercise timing or exercise intensity modulate the exercise benefits on motor learning (Roig et al., 2016; Thomas et al., 2016a, b). However, little is known regarding the modulatory effect of exercise duration. In adults, most studies examining the effect of moderate exercise on motor learning have used diverse protocols with durations of 30 min (Statton et al., 2015; Snow et al., 2016). In contrast, the majority of the studies analyzing the effect of IE have used exercise protocols of shorter durations (between 13 and 15 min of interval exercise) (Roig et al., 2012; Mang et al., 2014; Ferrer-Uris et al., 2017). To the best of our knowledge, no study has assessed the effect of an IE bout shorter than 12 min, or the modulatory effect of different exercise durations on motor learning in adults, and much less in a pediatric population.

Unlike the literature void regarding motor learning, previous research has studied the effect of short exercise bouts on cognitive learning, which may guide predictions in the motor learning domain. In adults, two 3-min sprints enhanced the acquisition and retention (exploratory comparisons) of a novel vocabulary task (Winter et al., 2007). In children, Etnier et al. (2014) observed improvements in a novel vocabulary task and its recall after performing a graded maximal running test lasting 9.88 min on average (ranging between 6.42 and 20 min). Moreover, some school-based exercise strategies of 10 min or less have obtained positive effects on children’s attention (Mahar et al., 2006; Ma et al., 2014). Therefore, short exercise bouts could show some promising results in enhancing motor learning.

Here, we investigated the effect of two IE intervention durations (short IE: a short bout of 5 min, and long IE: a long bout of 13 min) on the adaptation and retention of a motor task, which was a rotational visuomotor adaptation task (rVMA). We hypothesized that (1) IE would not enhance the adaptation of the rVMA, (2) the long IE would enhance consolidation of the rVMA, and (3) the short IE would also improve consolidation of the rVMA, although presenting a smaller effect compared to the long IE.

## Materials and Methods

Seventy-one children (40 male and 31 female, 9.13 ± 0.8 years) were recruited from two schools, one located in Barcelona, Spain (Site 1) and another in Los Angeles, United States (Site 2) (Table 1). Participants were excluded when they presented one or more of the following attributes: prior experience with the rVMA; left-handedness; low physical activity engagement; body mass index (BMI) above obesity threshold; intellectual quotient (IQ) below average score; parent-reported history of neurological, psychiatric, and/or physical impairment; non-corrected 20/20 vision; or current intake of medications affecting the nervous system and/or the ability to learn. By design, participants at Site 2 were randomly assigned to the experimental groups: (LONG) participants exercised for 13 min before practicing the rVMA; (SHORT) participants exercised for 5 min before practicing the rVMA; and (CON) who rested before engaging in the rVMA. At Site 1, participants were assigned to LONG or CON. Participants’ random distribution was separately checked at each site using age and fitness level as stratification factors because it has been reported that these two factors may impact the exercise effect on cognitive performance (Labelle et al., 2013), as well as the development of visuomotor representations (Contreras-Vidal et al., 2005). Identical materials and data collection and reduction procedures were used in both sites. However, group distribution was different by design.

**TABLE 1:** Group characteristics.

	**LONG**			**SHORT**	**CON**		
	**Site 1**	**Site 2**	**Site 1 + 2**	**Site 2**	**Site 1**	**Site 2**	**Site 1 + 2**
*N*	10	17	27	19	11	14	25
Sex (male/female)	7/3	8/9	15/12	11/8	6/5	9/5	15/10
Age (years)	9.16 ± 1.1	9.28 ± 0.7	9.24 ± 0.9	9.19 ± 0.9	8.78 ± 0.7	9.10 ± 0.6	8.96 ± 0.7
Height (cm)	135.66 ± 8.7	138.06 ± 7.2	137.17 ± 7.7	135.97 ± 7.6	135.45 ± 6.4	132.51 ± 5.1	133.80 ± 5.8
Body mass (kg)	32.90 ± 7.3	32.61 ± 6.8	32.71 ± 6.9	34.85 ± 8.5	32.82 ± 8.0	29.78 ± 0.7	31.11 ± 7.4
BMI (kg/m^2^)	17.72 ± 2.5	17.04 ± 3.0	17.29 ± 2.8	18.65 ± 3.2	17.67 ± 2.6	16.90 ± 3.5	17.24 ± 3.1
Estimated VO_2_max (ml/kg/min)	50.90 ± 4.2	46.16 ± 2.0	47.91 ± 3.7	44.80 ± 2.9	51.19 ± 5.6	45.36 ± 2.9	47.93 ± 5.1

*LONG, long exercise bout group; SHORT, short exercise group; CON, no exercise group; BMI, body mass index.*

Written and informed consent from all participants’ guardians and written assent from participants were provided prior to initiating the study in accordance with the Declaration of Helsinki. The study was approved by the Ethics Committee of Clinic Researches of the Catalan Sport Administration, and the Committee for the Protection of Human Subjects of the California State University, Northridge, CA, United States.

### Procedure

**FIGURE 1:**
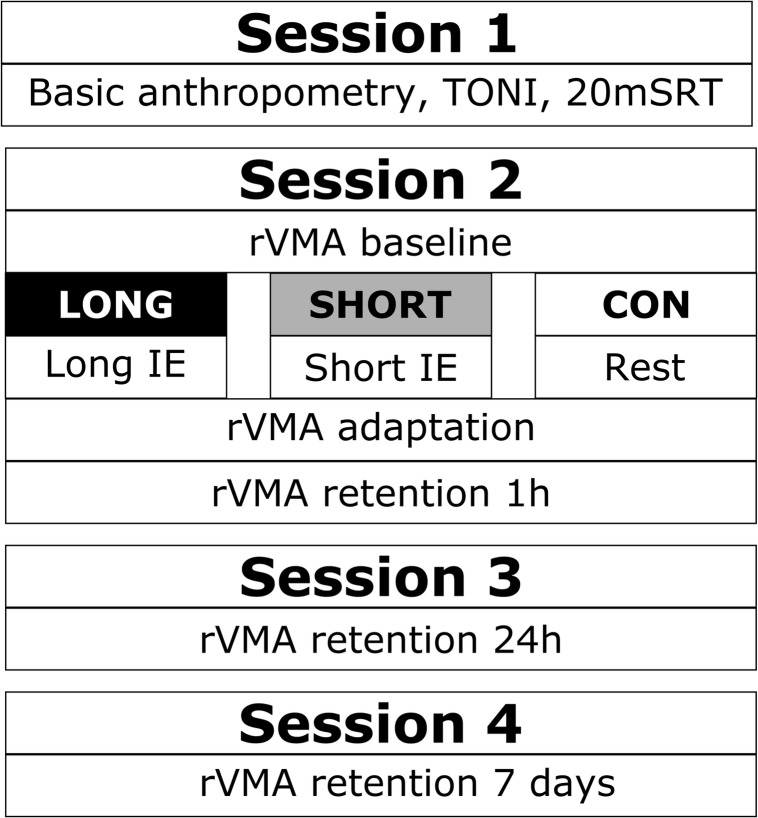
Study procedure. Schematic overview of the procedure of the four study sessions. TONI, test of non-verbal intelligence; 20mSRT, 20-meter shuttle-run test; rVMA, rotational visuomotor adaptation task; LONG, 13-min long exercise bout before the rVMA task; SHORT, 5-min short exercise bout before the rVMA task; CON, no exercise before the rVMA task; IE, intense exercise.

A total of four sessions were completed by each participant (Figure 1). Prior to the start of the first study session, guardians and children completed the Physical Activity Questionnaire for Children (PAQ-C) (Kowalski et al., 1997; Benítez-Porres et al., 2016) and the Physical Activity Readiness Questionnaire for children.

In Session 1, basic anthropometric characteristics (height and weight), IQ, and fitness level (estimated VO_2_max) of the children were assessed. IQ was assessed using the latest available version of the Test of Non-verbal Intelligence (TONI) (version 4 for the American sample and version 2 for the Spanish sample). Fitness level was evaluated using the 20-m shuttle-run test (20mSRT) (Léger et al., 1988). Beat-by-beat values for the RR intervals were collected with a Polar RS800CX (Polar Electro) at 1000 Hz frequency. A young fit adult accompanied the children running beside them to help maintain the pace and provide encouragement. Additionally, children carried a pictured card from side to side of the field in every even-numbered lap. The collected cards were used in a memory game at the end of Session 2 (only as incentive).

Forty-eight hours after Session 1, participants started Session 2 by being familiarized (20 trials) with the rVMA without cursor rotation (0°). Following familiarization, a baseline set (104 trials) was conducted to assess initial rVMA performance without cursor rotation (0°). Afterward, participants in both exercise groups engaged in the IE. To ensure equal test timing among groups between baseline and the next rVMA set, SHORT rested for 8 min before the IE warm-up. The transition time between the IE and the next rVMA set was 4 min for both exercise groups. Participants in CON had a rest period of 25 min while other participants exercised. After the IE or the rest period, participants engaged in the rVMA adaptation set (AD, 312 trials) where a 60°clockwise rotation was applied to the cursor movement. After a 1-h rest, all participants performed a second set of the 60° rotated rVMA. This was the first retention set (RT1h, 104 trials).

At the end of Session 2, all participants played the memory game with the cards they collected during the 20mSRT and the IE. During rest periods, participants were allowed to read or hold a conversation and were prevented from performing additional exercise or musical activity. During the third and fourth sessions, participants performed an additional set of the 60° rotated rVMA, 24 h and 7 days (RT24h and RT7d, 104 trials each) after the AD set.

### Rotational Visuomotor Adaptation Task

The rVMA task was conducted in a quiet room, where participants were seated 1 m away from a 19-inch screen. Participants controlled a screen cursor using a joystick. Cursor movement coordinates were registered at 120 Hz using an A/D NI-6008 card (National Instruments Corporation). A new target randomly appeared every 2 s in one of eight possible positions. Participants were instructed to run the cursor over the target doing one single move “as fast and as straight as possible” and then to move back to the home position (screen center).

### Intense Exercise

Two different IE protocols were programed: long IE and short IE. Both IE procedures were similar to a 20mSRT but with specific running intensities and volumes. During both exercise bouts, two speeds based on a percentage of the estimated VO_2_max were combined: a fast-paced speed (85% of VO_2_max) and a slow-paced speed (60% VO_2_max). The long IE (13 min total) interspersed three series of 3 min of fast speed with two series of 2 min of slow speed. The short IE (5 min total) consisted of two series of 2 min of fast speed separated by 1 min of slow speed. Before the start of the IE, participants of both exercise groups underwent a warm-up protocol consisting of 2 min running at slow speed and 1 min of fast speed. The warm-up was used to familiarize the participants with the running speeds of the IE. A 5-min rest period was defined between the warm-up and the IE. Participants’ heart rate was captured following the same procedure described in the 20mSRT. Heart rate data for the 85% of VO_2_max series (Table 2) indicated similar intensities across exercise groups. To encourage participants’ effort, an adult also accompanied them during the exercise, and they continued collecting memory game cards during the IE.

**TABLE 2:** Group average and standard deviation heart rate data of the last 1/6th of each 85% VO_2_max series.

	**Serie 1 (bpm)**	**Serie 2 (bpm)**	**Serie 3 (bpm)**	**Grand mean**
LONG	185.0 ± 13.8	189.5 ± 14.4	190.0 ± 17.7	188.4 ± 13.8
SHORT	183.5 ± 20.3	186.2 ± 19.0	NA	184.8 ± 19.5

**T*-test of the 85% VO_2_max. Grand mean: *t*_(__42__)_ = 0.695, *p* = 0.491. LONG, long exercise bout group; SHORT, short exercise group; bpm, beats per minute.*

### Data Reduction

Custom MATLAB R2014b programs (The MathWorks, Inc.) were used to reduce the Cartesian positions of the joystick movement. Data were low-pass filtered using an eight-order dual-pass Butterworth filter with a cut-off frequency of 12 Hz. Accepted trials fulfilled the following conditions: startup position found within 20% of the target radius (distance from center to target), and total traveled distance from the screen center to the end of the movement was equal to or greater than 90% of the target radius. For the accepted trials, the nearest point to an outward movement equal to 10% of the target radius distance was defined as the movement onset. Movement offset was defined as the point where the speed of the movement decreased to 10% of its maximum value. The AD set was divided in epochs of eight trials for further analyses.

### Variables

**FIGURE 2:**
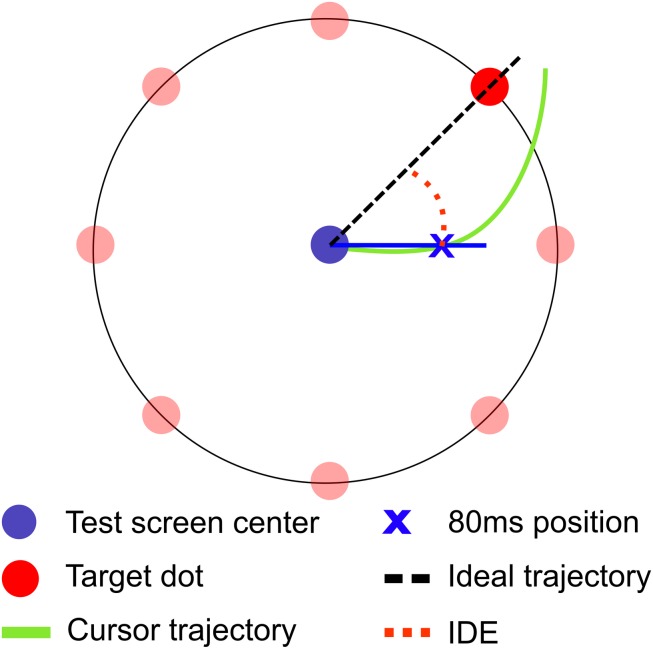
Rotational visuomotor adaptation task (rVMA) and initial directional error (IDE) variable. The center dot is the starting home position for each trial. The remaining dots are the targets which appeared every 2 s in one of eight possible locations (45, 90, 135, 180, 225, 270, 315, and 360°) and remained visible during 750 ms. To compute the IDE, two movement trajectories were defined: the ideal trajectory and the real initial trajectory. Ideal trajectory was defined as the linear vector from the center of the test screen to the target. The real initial trajectory was defined as the linear vector from the test screen center to the cursor position 80 ms after the movement onset. IDE stands for the output error before visual feedback was available to correct the cursor trajectory, so IDE was an indirect measure of the motor planning of the movement.

Movement descriptive characteristics were: movement time (MT, ms), travel distance (TD, cm), and reaction time (RT, ms). MT and TD were defined as the time and the displacement, respectively, between movement onset and offset. RT was defined as the time window from the target appearance to the movement onset. The initial directional error (IDE, degree) was computed as a representative of output error (Figure 2). IDE was calculated as the angular difference between two linear vectors, representing the ideal movement trajectory and the real initial trajectory from the home position to the cursor position at 80 ms after movement onset. IDE was used as a measure of the rotation adaptation or motor memory consolidation, avoiding the possible trajectory correction through perceptual feedback (Contreras-Vidal et al., 2005). IDE is considered a good estimate of movement planning and, thus, of the formation of the internal model of the motor skill (Contreras-Vidal et al., 2005; King et al., 2009; Kagerer and Clark, 2014). Moreover, this variable was utilized as our main outcome because IDE has been observed to be particularly sensitive to exercise effects (Ferrer-Uris et al., 2017, 2018). Considering the large inter-subject variability usually presented by children, computed adaptation and retention variables were normalized by the participant’s baseline mean values.

Initial directional error followed a decay flow during AD set that resulted to be best fitted by a double exponential function, as seen in previous research (Krakauer et al., 2005). The error decay presented an initial rapid reduction followed by a slower decay of the error. The initial rate of learning (RL) is a measure of the initial rapid error reduction. Individual RL was computed for IDE (RL-IDE) as the first derivative of the first half of the double exponential function and evaluated at epoch 1, similarly as described in Coats et al. (2014).

### Data Analysis

Normality distribution was checked for all variables via histograms and the Kolmogorov–Smirnov’s normality test. Variable transformation or non-parametric alternative tests were used when necessary (baseline MT and baseline IDE were transformed). To control for the group distribution differences across sites, we implemented a four-step process: (1) possible site differences at baseline were explored through Student’s *t*-test or the Mann–Whitney *U* test comparing age, estimated VO_2_max, and the rVMA baseline variables (MT, TD, RT, and IDE); (2) to examine possible group differences at baseline, age, and estimated VO_2_max and rVMA baseline variables were explored through one-way analysis of variance (ANOVA); (3) we studied the association between the IDE variable and those variables that presented baseline site or group differences, using the Pearson correlation coefficient; and (4) those variables that presented significant site or group differences and significant correlation with the IDE were used as covariates in the subsequent analyses. For the first study hypothesis, where the effect of the IE bout on the AD of the rVMA task was examined, mean values of the AD error variables (IDE and RL-IDE) were compared using one-way analysis of covariance (ANCOVA) with group as the independent factor. The second and third hypotheses, related to the effect of exercise on motor retention and the modulation effect of the exercise length on the retentions of the rVMA, were explored with two-way (Group × Set) repeated measures ANCOVAs, comparing the mean IDE retention values. Greenhouse–Geisser sphericity-corrected values were reported when appropriate. Bonferroni-corrected *post hoc* tests were performed when significant differences were found.

The effect size of the different tests was calculated according to Cohen (1988): *d* for *t*-test (0.2 small, 0.5 medium, and 0.8 large effect), *r* for Mann–Whitney *U* test (0.1 small, 0.3 medium, and 0.5 large effect), and $\eta_{\text{p}}^{2}$ for ANOVAs and ANCOVAs (0.01 small, 0.06 medium, and 0.14 large effect). Statistical significance was set at *p* < 0.05 for all comparisons.

## Results

The analysis of the possible site effect on the participants’ characteristics showed that participants from Site 1 (BCN) presented higher VO_2_max [*t*_(__69__)_ = 5.026; *p* < 0.001; *d* = 1.442], compared to the participants in Site 2 (LA). When the site effect was explored for the rVMA baseline variables, we found significant statistical differences between sites for the MT [*t*_(__69__)_ = 8.331; *p* < 0.001; *d* = 2.164], TD [*t*_(__69__)_ = 6.496; *p* < 0.001; *d* = 1.786], and RT [*t*_(__69__)_ = 2.979; *p* < 0.001; *d* = 0.805] variables, indicating that participants from Site 1 executed the task with longer MT, and shorter TD and RT. However, IDE, which does not directly depend on the movement approach but on the planning of the movement, showed no differences between sites [*t*_(__69__)_ = 0.342; *p* = 0.733; *d* = 0.160].

Participants’ characteristics and the average rVMA baseline performance were analyzed via ANOVAs to check main group effects. No group differences were found regarding participant characteristics (age and VO_2_max). Similarly, no group differences were observed regarding rVMA baseline performance (MT, TD, RT, and IDE; see Table 3 for means and SD). Therefore, groups presented a balanced distribution in terms of age, VO_2_max, and rVMA baselines performance despite the aforementioned site effects.

**TABLE 3:** Mean and SD performance values on the rotational visuomotor adaptation task (rVMA) for each group, site, and set.

	**LONG**			**SHORT**	**CON**		
**Sets**	**Site 1**	**Site 2**	**Site 1 + 2**	**Site 2**	**Site 1**	**Site 2**	**Site 1 + 2**
**Baseline**							
MT (ms)	196.15 ± 28.64	150.36 ± 23.47	167.32 ± 33.62	145.72 ± 14.71	195.72 ± 26.41	143.61 ± 13.70	166.53 ± 33.00
TD (cm)	6.85 ± 0.32	7.57 ± 0.45	7.30 ± 0.53	7.35 ± 0.46	6.67 ± 0.36	7.53 ± 0.46	7.15 ± 0.59
RT (ms)	404.15 ± 36.97	439.85 ± 37.90	426.63 ± 40.81	433.24 ± 46.66	419.14 ± 32.54	459.93 ± 37.16	441.98 ± 40.20
IDE (deg)	12.44 ± 6.08	10.40 ± 2.36	11.15 ± 4.15	11.76 ± 6.23	10.67 ± 3.26	9.96 ± 2.10	10.27 ± 2.63
**Adaptation**							
MT (ms)	199.04 ± 26.7	145.96 ± 20.36	165.62 ± 34.41	146.00 ± 18.38	214.39 ± 38.35	151.75 ± 17.52	179.31 ± 42.26
TD (cm)	7.00 ± 0.31	7.75 ± 0.38	7.74 ± 0.51	7.52 ± 0.45	6.83 ± 0.27	7.79 ± 0.18	7.37 ± 0.53
RT (ms)	408.23 ± 46.82	455.30 ± 38.84	437.87 ± 47.16	451.73 ± 47.45	423.11 ± 45.03	456.94 ± 32.60	442.06 ± 41.41
IDE (deg)	28.67 ± 6.01	30.44 ± 8.74	29.78 ± 7.77	30.08 ± 7.62	32.38 ± 7.94	32.811 ± 0.18	32.62 ± 9.08
RL_IDE	−4.09 ± 4.65	−5.16 ± 3.29	−4.77 ± 3.77	−6.11 ± 6.29	−6.10 ± 5.74	−4.29 ± 2.19	−5.10 ± 4.12
**Retention 1 h**							
MT (ms)	204.79 ± 30.48	142.57 ± 16.32	165.61 ± 37.72	133.75 ± 13.61	193.51 ± 31.14	140.47 ± 19.54	163.81 ± 36.51
TD (cm)	7.06 ± 0.34	7.91 ± 0.32	7.60 ± 0.53	7.91 ± 0.42	7.00 ± 0.31	7.90 ± 0.29	7.50 ± 0.54
RT (ms)	418.07 ± 45.40	448.73 ± 43.72	437.38 ± 46.01	443.98 ± 53.11	432.09 ± 41.01	451.63 ± 35.82	443.03 ± 38.65
IDE (deg)	17.43 ± 3.40	18.73 ± 6.14	18.25 ± 5.26	20.16 ± 7.01	24.041 ± 0.14	22.35 ± 7.00	23.09 ± 8.37
**Retention 24 h**							
MT (ms)	211.88 ± 33.80	143.97 ± 17.56	169.12 ± 41.25	137.37 ± 16.46	194.72 ± 31.35	143.77 ± 22.78	166.19 ± 36.84
TD (cm)	7.16 ± 0.35	7.82 ± 0.27	7.58 ± 0.44	7.89 ± 0.37	7.27 ± 0.45	7.75 ± 0.29	7.54 ± 0.44
RT (ms)	414.44 ± 48.04	459.42 ± 43.60	442.76 ± 49.58	444.00 ± 51.22	427.44 ± 30.95	450.71 ± 41.64	440.47 ± 38.44
IDE (deg)	17.02 ± 4.28	18.94 ± 5.40	18.23 ± 5.02	18.76 ± 5.71	23.49 ± 7.42	20.85 ± 4.28	22.01 ± 5.89
**Retention 7 days**							
MT (ms)	203.87 ± 31.36	144.63 ± 20.02	166.57 ± 37.91	133.67 ± 16.12	195.37 ± 33.96	137.12 ± 11.90	162.75 ± 37.79
TD (cm)	7.30 ± 0.31	7.81 ± 0.39	7.62 ± 0.44	7.79 ± 0.32	7.27 ± 0.34	7.77 ± 0.35	7.55 ± 0.42
RT (ms)	406.93 ± 51.39	445.37 ± 44.39	431.14 ± 49.85	431.25 ± 46.36	422.40 ± 42.6	443.42 ± 30.53	434.17 ± 37.07
IDE (deg)	17.72 ± 4.41	17.20 ± 5.59	17.39 ± 5.10	19.17 ± 5.07	21.32 ± 7.90	20.97 ± 6.20	21.12 ± 6.84

*LONG, long exercise bout group; SHORT, short exercise group; CON, no exercise group; MT, movement time; TD, travel distance; RT, reaction time; IDE, initial directional error; RL, rate of learning.*

Correlation analysis between IDE and those variables presenting site differences at baseline (MT, TD, RT, and estimated VO2max) was performed using the Pearson correlation coefficient. IDE presented low but significant correlations with the MT (*r* = 0.255; *p* = 0.032) and the RT (*r* = −0.411; *p* < 0.001) variables. Therefore, baseline MT and RT were used as covariates in the subsequent analyses.

**FIGURE 3:**
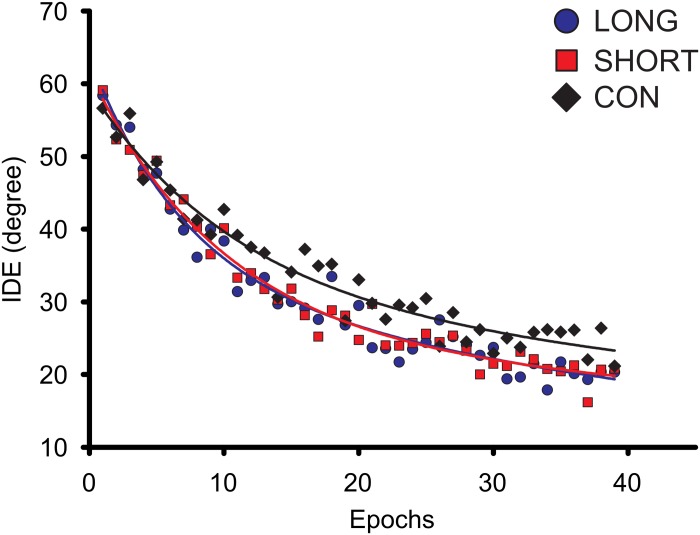
Group average learning curves for the raw initial directional error (IDE) during the adaptation set. Each data point represents the average of eight consecutive trials (one epoch). LONG, long exercise bout before the rVMA task; SHORT, short exercise bout before the rVMA task; CON, no exercise before the rVMA task.

**FIGURE 4:**
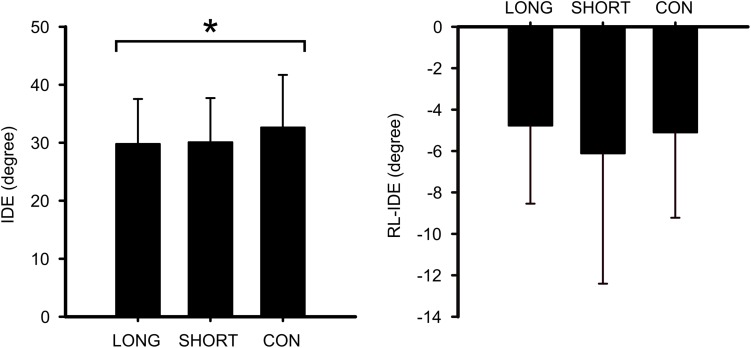
Group average error and rate of learning during the adaptation set by group. Mean and standard deviations of the initial directional error (IDE) and the rate of learning (RL-IDE) during the adaptation set of the rotational visuomotor adaptation task (rVMA) are presented. Normalized IDE and RL-IDE values are compared among groups. A significant main group effect is represented by (^∗^). LONG, long exercise bout before the rVMA task; SHORT, short exercise bout before the rVMA task; CON, no exercise before the rVMA task.

To examine the possible exercise effect on the adaptation of the rVMA task, we explored the effect of the SHORT, LONG, and CON interventions on the average IDE of the adaptation set, as well as the initial RL of the IDE variable (RL-IDE) (Figure 3). The ANCOVA revealed group differences regarding IDE [*F*(_2_,_68_) = 3.160; *p* = 0.049; $\eta_{\text{p}}^{2}$ = 0.087] (Figure 4). However, no statistical differences were found between groups. Moreover, no significant group differences or covariate effects were found for RL-IDE. In addition, the covariate MT, but not RT, was found to be significant in the IDE analysis [*F*(_2_,_68_) = 7.1600; *p* = 0.009; $\eta_{\text{p}}^{2}$ = 0.098].

**FIGURE 5:**
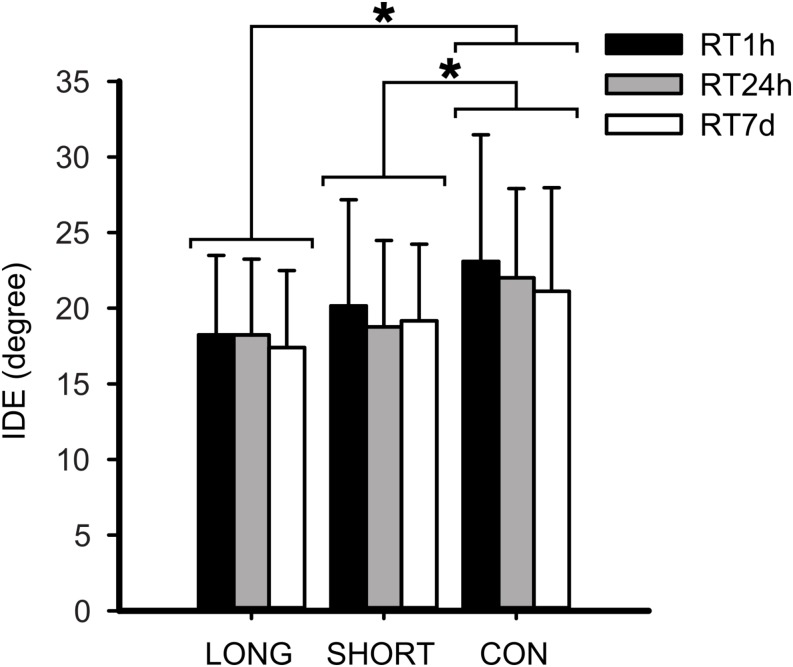
Mean and standard deviation for the rotational visuomotor adaptation task (rVMA) error performance by group during retention. Normalized error values for the average retention initial deviation error (IDE) are compared by group. Significant differences are represented by (^∗^). LONG, long exercise bout before the rVMA task; SHORT, short exercise bout before the rVMA task; CON, no exercise before the rVMA task.

The effects of the IEs on the retention sets of the rVMA task were analyzed using a repeated measure ANCOVA. A significant group effect was found for the IDE [*F*(_2_,_68_) = 6.108; *p* = 0.004; $\eta_{\text{p}}^{2}$ = 0.156]. *Post hoc* analysis indicated that both exercise groups had lower IDE values in comparison to CON (LONG: *p* = 0.002; *d* = 0.868, SHORT: *p* = 0.022; *d* = 0.708) (Figure 5). No IDE difference was observed between the two exercise groups (SHORT and LONG). Furthermore, no covariate turned out to be significant. In addition, no significant main set effect or Group × Set interaction effect were observed.

## Discussion

The main focus of this study was to observe the effect of two exercise protocol durations (5 min, short bout, and 13 min, long bout) on the adaptation and retention of a rVMA using IDE as the main outcome variable.

Regarding motor adaptation, previous research suggested that IE may produce a null effect on adaptation in adults (Ferrer-Uris et al., 2017) and children (Ferrer-Uris et al., 2018), possibly because of inducement of fatigue (Roig et al., 2012) or excessive arousal (McMorris and Hale, 2012). We hypothesized that both long and short IE would not affect motor adaptation. According to our hypothesis, all groups presented similar performance during the adaptation set. The results of the present study add new evidence and support the previous evidence about the exercise-induced effects on motor learning, suggesting that IE may not improve motor adaptation even when exercise duration is short. However, more research may be necessary to further speculate on this topic, especially considering that a main group effect during adaptation was found, though it could not be confirmed during the pairwise group comparisons. More studies focusing on the analysis of the underlying mechanisms regarding exercise-induced benefits under different exercise intensity and duration conditions, both in adults and children, are needed. Furthermore, our results should be interpreted with caution considering the asymmetrical group distribution in this study.

Regarding motor retention (as an estimate of the motor memory consolidation process) and based on previous research using similar long exercise bouts to enhance motor memory consolidation in adults (Roig et al., 2012; Ferrer-Uris et al., 2017) and children (Lundbye-Jensen et al., 2017; Ferrer-Uris et al., 2018), we hypothesized that LONG would enhance motor memory consolidation. Furthermore, based on the evidence that intense short exercise bouts have facilitated declarative learning in adults (Winter et al., 2007) and children (Etnier et al., 2014), we hypothesized that, although LONG would present a greater effect, SHORT would also improve motor memory consolidation. Results indicated that the movement planning of the motor skill (IDE) improved during the retention sets presumably as a result of the exercise interventions. Interestingly, this effect seemed to take place already 1 h from adaptation and lasted for 7 days. However, further research would be necessary to examine if motor learning form (e.g., motor adaption or motor sequence learning) may impact the time course of the motor consolidation process. To the best of our knowledge, this is the first piece of evidence of a facilitation of motor memory consolidation after a bout of exercise as short as 5-min duration. These exercise-based improvements cannot be solely explained by a site effect since differences were not found between sites for the IDE variable at baseline and LONG was composed by participants from both sites. In addition, when looking at the movement descriptive variables (Table 3), it appears that groups presented a similar movement approach during retentions, which indicates that IDE differences among groups may not be explained by the movement approach, but by the participants’ movement planning. Moreover, IDE group differences during retention were not related to any of the controlled variables.

Previous research in adult populations has proposed that exercise benefits on consolidation of declarative memory (Winter et al., 2007; Segal et al., 2012) and motor memory (Mang et al., 2014; Skriver et al., 2014) may be produced by a stimulation of the learning-related neuroplasticity mechanisms. Neurotrophins, such as brain-derived neurotrophic factor, and catecholamines (epinephrine, norepinephrine, and dopamine), are some of the biomarkers that are usually associated with enhancements in synaptic plasticity (Taubert et al., 2015). Intense physical exercise has been related to increases in peripheral blood concentrations of these neurochemical compounds (Winter et al., 2007). Increases in blood lactate concentration have also been seen to positively correlate with improvements in motor memory consolidation (Skriver et al., 2014). Even though a causal relation between lactate and synaptic plasticity may be difficult to ascertain, it is thought that lactate could act as intermediary in the synaptic plasticity mechanisms, possibly involved in growth-factor signaling and brain energetics (Skriver et al., 2014; Taubert et al., 2015). However, these relations have to be interpreted with caution because of the difficulties of measuring central concentration of these biomarkers in humans, thereby limiting the establishment of a causal link between exercise, neurochemicals, and consolidation. Moreover, despite some evidence regarding exercise-induced catecholamine increases (Wigal et al., 2003), little is known of these mechanisms

in children. In addition to this evidence, one should also consider exercise-increased activation of certain brain areas as a potential underlying mechanism for the benefits of exercise in relation to learning a motor task (Rajab et al., 2014; Stavrinos and Coxon, 2016). Previous evidence revealed how IE promoted a sustained increase in corticospinal excitability, which was related to the motor skill performance off-line improvements obtained during memory consolidation (Ostadan et al., 2016). Moreover, another recent study demonstrated that exercise-modulated brain activity, functional connectivity and cortico-muscular coherence, improved motor memory consolidation of a motor skill when compared to a non-exercise control group (Dal Maso et al., 2018). However, at present we have no available information regarding brain activity related to exercise and motor learning in children. Therefore, further research is required to explore the potential mechanisms underlying the existing beneficial relation between exercise and motor memory consolidation, especially in children.

This study presents new insights about the influence of exercise duration on the consolidation of procedural memory in children. The results in the present study suggest that IE can enhance children’s motor learning (mostly consolidation), even with bouts as short as 5 min. Previous studies demonstrated benefits in children’s attention when performing short exercise sessions during school hours (Mahar et al., 2006; Ma et al., 2014). Therefore, we think that short IE bouts could be helpful in school or sport environments, where children engage in the learning of many new motor skills. Whether similar results can be found with different types of exercise should be explored in future research, given that different exercise types yielded positive motor memory consolidation in adults (Thomas et al., 2017). The limitations of our study include: difficulties to generalize exercise benefits to other motor skills or forms of learning, missing a comparison group where exercise was performed after the adaptation set, relatively small sample, and initial differences between sites. Despite these limitations, we argue that IE with durations of 5 or 13 min could improve motor memory consolidation. Considering previous evidence regarding the moderator effect of exercise intensity, along with the present study results, one could consider the possible dose–response relation between exercise and motor learning enhancements, a topic that should be more thoroughly addressed in future research.

## Conclusion

Retention of the rVMA was enhanced through acute exercise in children. These data suggest that motor memory consolidation was improved even when the exercise duration was only 5 min. As expected, IE bouts did not improve the adaptation of the motor task, even when exercise duration was short. The presented exercise benefits on motor memory consolidation in children could support the use of acute IE bouts in school and sport environments.

## Data Availability

All datasets generated for this study are included in the manuscript and/or the supplementary files.

## Ethics Statement

Written consent from all participants’ guardians and written assent from participants were provided prior to initiate the study in accordance with the Declaration of Helsinki. The study was approved by the Ethic Committee of Clinic Researches of the Catalan Sport Administration and the Committee for the Protection of Human Subjects of the California State University, Northridge, CA, United States.

## Author Contributions

All authors participated in the study conceptualization, data collection and analysis, and manuscript preparation and revision, and contributed equally to this study.

## Conflict of Interest Statement

The authors declare that the research was conducted in the absence of any commercial or financial relationships that could be construed as a potential conflict of interest.
